# CD137L-macrophage induce lymphatic endothelial cells autophagy to promote lymphangiogenesis in renal fibrosis

**DOI:** 10.7150/ijbs.66781

**Published:** 2022-01-01

**Authors:** Haotian Wei, Li Chen, Qing Li, Xinjun Liang, Kun Wang, Ying Zhang, Yueqiang Li, Yanyan Liu, Gang Xu

**Affiliations:** 1 Department of Nephrology, Division of Internal Medicine, Tongji Hospital, Tongji Medical College, Huazhong University of Science and Technology, Wuhan, Hubei, China; 2 Department of Medical Oncology, Hubei Cancer Hospital, Tongji Medical College, Huazhong University of Science and Technology, Wuhan, Hubei, China; 3 Department of Nephrology, Xiaogan Central Hospital, Xiaogan, Hubei, China

**Keywords:** CD137L, macrophage, autophagy, lymphangiogenesis, renal fibrosis

## Abstract

Renal lymphangiogenesis is a new field of international nephrology in recent years and plays an important role in the progression of chronic renal disease. CD137 was originally described as a surface molecule present on activated T and NK cells and detected on hypoxic endothelial cells and inflamed blood vessels, but its function on lymphatic endothelial cells remains unclear. We investigated the relationships among CD137, lymphangiogenesis and macrophages, which are involved in interstitial fibrosis. Similar to other chronic inflammatory diseases, we found lymphangiogenesis and expression of CD137 in the renal tissue of patients with IgA nephropathy. CD137-positive lymphatic vessels were involved in the development process of IgA nephropathy and positively correlated with serum creatinine, serum urea nitrogen, serum uric acid, and urinary 24 h total protein. The expression of these indicators was negatively correlated with eGFR, plasma albumin, and HB. In mouse models of UUO, we verified that CD137 expression was significantly elevated during lymphangiogenesis and that its ligand CD137L was released by macrophages after VEGF-C stimulation in the kidney. In vitro, recombinant CD137L significantly enhanced LEC proliferation, migration and tube formation, and these effects were inhibited by CD137 siRNA. Mechanistically, the CD137L interaction with CD137 induced the transition from LC3-I to LC3-II and the expression of Atg5, Atg7, Atg12 and p62 proteins by activating the PI3K/AKT/mTOR pathway to promote autophagy. Knockdown of Atg5 and Atg7 blocked CD137L-induced autophagy. Thus, we propose that CD137L secretion by macrophages interacts with CD137 on lymphatic endothelial cells to prompt lymphangiogenesis in the kidney, which further drives fibrogenic responses. Our findings suggest that inhibition of the CD137-CD137L pathway is a novel therapeutic approach for obstructive nephropathy.

## Introduction

Renal fibrosis (RF) is the final common pathway of all chronic kidney diseases, especially the progression of IgA nephropathy (IgAN), leading to a gradual loss of renal function. In the stage of renal fibrosis, humoral immunity, hypoxia, proteinuria and acute injury cause parenchymal cell damage and the rapid recruitment of several inflammatory cells to the kidney 1-3. Then, the damaged parenchymal cells and the inflammatory cytokines and chemokines induced more different types of inflammatory cells to the lesion site. These inflammatory cells secrete a large number of cytokines and growth factors to upregulate the differentiation of renal tubular epithelial cells and endothelial cells to mesenchymal cells, accelerate the recruitment of bone marrow myofibroblasts, promote the activation and proliferation of inherent fibroblasts in the kidney, and lead to the development of renal fibrosis 4-6. Thus, inflammatory cells play an important “push role” either in the initial stage of renal fibrosis or in progress 7.

Evidence shows that the complex processes of inflammation are closely associated with lymphangiogenesis in some pathological conditions 8-11. Lymphangiogenesis, as an immune-related factor, regulates inflammatory cells in different organs. In inflammatory bowel disease, an increase in lymphangiogenesis indicates a good prognosis, while other forms of lymphatic alterations, including lymphangiectasia, lymphadenopathy, and lymphatic vessel occlusion, denote poor prognosis 12. During cancer progression in particular, lymphangiogenesis can exert both positive and negative effects 13. While the formation of tumor-associated lymphangiogenesis correlates with metastatic dissemination, increased severity and poor patient prognosis, the presence of functional lymphatics is regarded as beneficial for antitumor immunity and cancer immunotherapy delivery. Similarly, traditional opinion holds that lymphangiogenesis is good for clearing accumulated fluid and immune cells in kidney disease 14. The latest studies have provided insights into the role of lymphangiogenesis, which may propagate an inflammatory feedback loop, aggravating inflammation and fibrosis. Despite the explosion of knowledge on the role of lymphatic biology, several gaps remain in our understanding of the function of lymphangiogenesis in renal fibrosis.

CD137 has been shown to play a key role in T-cell-mediated responses and acts as a costimulatory molecule by binding to its ligand (CD137L) 15-17. CD137 on the surface of T helper type 1 cells (Th1) binds to CD137L expressed by macrophages or DCs and activates nuclear factor κB (NF-κB) by signal transduction, leading to the activation of macrophages or the maturation of DCs 18-20. Recent studies have shown that the interaction of CD137-CD137L is involved in the regulation of multiple stages of the inflammatory response and plays an important role in the cascade amplification of inflammatory responses 21-22. In renal inflammatory microenvironments, whether the CD137-CD137L signaling pathway is involved in the maintenance of inflammation and promotion of lymphangiogenesis is unknown. In this study, we aimed to identify the roles of CD137L secreted by macrophage in the development of lymphangiogenesis in a unilateral ureteral obstruction (UUO) model. In addition, we studied the relationships among CD137^+^ lymphatic vessels, CD137L on macrophages, and enhanced autophagy, which are all involved in interstitial fibrosis.

## Materials and methods

### Patients

Renal biopsy specimens were obtained from 85 patients with IgA nephropathy diagnosed between January 2020 and April 2020 in the Division of Nephrology, Tongji Hospital, Tongji Medical College, Huazhong University of Science and Technology. All patients met the diagnostic criteria of the Oxford Classification of IgA nephropathy 2016 23. Patients were excluded from this study if they met the following conditions: <18 years of age, had a concomitant autoimmune disease, pregnancy, presence of active infection and received glucocorticoid or immunosuppressant treatment before renal biopsy. Our protocol was approved by the institutional review board or ethics committee at each center. Written informed consent was obtained from all patients.

### Mice

Male C57BL/6 mice (age, 6-8 weeks; weight, 20-25 g) were purchased from Beijing Vital River Laboratory Animal Center (Beijing, China). All mice were bred and maintained in specific pathogen-free (SPF) conditions at Tongji Medical College of Huazhong University of Science and Technology. Mice were anesthetized and euthanized with 1% sodium pentobarbital solution (0.009 ml/g, Sigma, USA) by i.p. injection. The renal fibrosis model was induced by unilateral ureteral obstruction (UUO) as previously reported. anti-CD137 blocking antibody (4-1BB, Clone:17B5, Bio X Cell, Lebanon NH) were injected (i.p.) at 10 mg/kg/dose twice weekly.

### Immunohistochemistry and immunofluorescence

The tissues were fixed in 4% paraformaldehyde and embedded in paraffin. Antigen was recovered in citrate buffer PH 6. The slides were blocked with 3% H202 for 30 min and nonspecific antigens were blocked with serum for 30 min. Paraffin sections were were incubated with primary rabbit anti-CD137 antibody (1:100, Abcam, USA) at 4 °C overnight. The sections were then incubated with biotinylated goat anti-rabbit Ig antibody as the secondary antibody for 30 mins, and the antibody reactions were visualized using diaminobenzidine (Gene Tech, Shanghai, China). An irrelevant isotype mouse Ig was used as the negative control.

For immunofluorescence, the sections were fixed in stationary liquid (methanol, acetone 1:1) for 10 minutes and washed with PBST (0.3% Tween 20 in PBS) three times. After blocking with 5% goat serum (Zsbio, Beijing, China), the fixed tissues were incubated with the following primary antibodies: mouse anti-D2-40 antibody (Genetech, China) and rabbit anti-CD137 antibody (1:100, Abcam, USA) at 4 °C overnight. After washing with PBST, the sections were incubated with the following secondary antibodies: CY3-goat anti-rabbit IgG (1:100, Amyjet, China) and Alexa Fluor488-goat anti-mouse IgG (1:100, Amyjet, China). DAPI (1:300, Sigma-Aldrich, St Louis, MO) was used for nuclear staining. The samples were washed with PBST and mounted with fluorescence mounting medium (DAKO, Carpinteria, CA). Fluorescence signals were visualized, and digital images were obtained by a Nikon DXM 1200 digital camera and analyzed by Image-Pro Plus software in a blinded manner.

### Western blotting

Kidney tissues were lysed in RIPA buffer (25 mM Tris-HCl, 150 mM NaCl, 1% NP-40, 1% sodium deoxycholate, 0.1% SDS, complete protease cocktail (Roche)), incubated on ice for 30 min and then collected by centrifugation at 12,000 rpm. for 30 min. Approximately 20 ug of protein lysate boiled with loading buffer was separated with SDS-PAGE electrophoresis under reducing conditions and electrotransferred onto a polyvinylidene difluoride membrane (Millipore). Following blocking with 5% (w/v) low-fat milk in TBST (0.1% Tween-20 in TBS) for 1 h at room temperature, the membrane was incubated with primary antibodies diluted in blocking buffer overnight at 4 °C, followed by incubation with horseradish peroxidase-conjugated secondary antibodies for 1 h at room temperature. Western blotting was developed using enhanced chemiluminescence (ECL Plus; Pierce). The relative intensities of bands were quantified by ImageJ software.

### Flow cytometry

The kidney tissue was weighed and put into six-well plates, and the tissue was cut with scissors, 2 ml of 0.2% pancreatase and digested in a 37 ℃ thermostat for 45min. Tissue suspension was terminated by digestion with PBS with serum, filtrated by a strainer, and centrifuged at 1500 rpm. for 5 minutes 3 times. The cells were counted under a microscope, and the concentration of the cells in each tube was adjusted according to the count so that the cell suspension of each tube was 1 × 10^6^ cells. Primary surface antibody incubations occurred for 30 min at 4 °C. Isotype controls were added at the same concentration as primary antibodies. Samples were analyzed on a FACS Calibur (BD Biosciences) using Cell Quest software (BD Pharmingen).

The kidney tissues were weighed and put into six-well plates, and the tissues was cut with scissors and minced, digested in 2ml of collagenase I in a 37 ℃ thermostat for 45 min. Tissue suspension was terminated by digestion with PBS, filtrated by a strainer, and centrifuged at 1500 rpm. for 5 min. Cells were resuspended in 1 mL 1x red blood cell lysis buffer, incubated for 3 min, and washed out at 1500 rpm for 5 min with PBS. The cells were counted under a microscope, and the concentration of the cells in each tube was adjusted according to the count so that the cell suspension of each tube was 1 × 10^6^ cells. Primary surface antibody incubations occurred for 30 min at 4 °C. The following additional antibodies were obtained from BioLegend: APC/Cy7-conjuated anti-mouse CD45 antibody, FITC-conjugated anti-mouse CD11b antibody, APC-conjugated anti-mouse F4/80 antibody, PE-conjugated anti-mouse CD137L antibody, BV421-conjugated Zombie dye. Samples were analyzed on a FACS Calibur (BD Biosciences) using Cell Quest software (BD Pharmingen). The data was analyzed using Flowjo v10.4 software.

### Real-time PCR

For extraction of total RNA, cells were homogenized in 1 ml TRIzol reagent, and chloroform was added to the homogenates. The mixture was incubated for 10 min and centrifuged at 12,000 x rpm for 20 min at 4 ℃. Then, the chloroform was added into isopropanol. The mixture was incubated for 30 min and centrifuged at 12,000 x rpm for 25 min at 4 ℃. After resuspending the pullet in 75% ethanol, the mixture was centrifuged at 12,000 x rpm for 10 min twice. RNA concentration was measured by the absorbance at 260 nm and 280 nm. One microgram of RNA was used for reverse transcription with a PrimeScriptTM RT reagent kit (Takara, Tokyo, Japan). The cDNA template was mixed with SYBR green PCR mix (Takara). Real-time PCR was performed using a Roche Real-Time PCR system. The following primers were used: mouse CD137 forward, CGTGCAGAACTCCTGTGATAAC; mouse CD137 reverse, GTCCACCTATGCTGG AGAAGG; mouse CD137L forward, CTATGGCCTAGTCGCTTTGGT, mouse CD137L reverse, CTATGGCCTAGTCGCTTTGGT; mouse gapDH forward, AGGTCGGTGTGAACGGATTTG, mouse gapDH reverse, TGTAGACCATGTAG TTGAGGTCA.

### CCK8 assay

Cell proliferation was determined using the CCK-8 assay (MCE, USA). LECs were seeded and cultured in 96-well plates at a density of 10^3^/well in 100 μL medium. The cells were treated with recombinant CD137L 100 ng/mL and CD137 inhibitory for 24, 48, and 72 h. After treatment, 10 μL of CCK-8 reagent was added to each well and incubated for 2 hours away from light. The absorbance was measured at 405 nm using a microplate reader. All experiments were performed in triplicate.

### Electron microscopy

LECs were trypsinized and pellected. The cells were fixed with 2.5% glutaraldehyde at 4 ℃ for 24 h. After washing with PBS 3 times, the cells were fixed in 1% osmium tetroxide, dehydrated, and treated with propyltetrorxide, and then the cells were embedded in epoxy resin. Then, 100-nm sections were cut on an ultramicrotome and picked up on copper grids. The grids were poststained in uranyl acetate and bismuth subnitrate. Images were taken using an electron microscope (Hitachi, Japan).

### Cell migration assays

Transwell assays were performed using 24-well Transwell cell culture chambers (Corning Life Science). Briefly, LECs were seeded into the top chamber at a density of 2.5 x 10^5^/well in 100 μl of serum-free medium, and 750 μl of cultured medium with or without recombinant CD137L protein was added to the lower chamber. After incubation for 12 h, the migrated cells on the lower membrane surface were fixed with 4% paraformaldehyde and then stained with crystal violet. Five random fields were counted per chamber by an inverted microscope (Olympus, Japan). Each experiment was repeated three times.

### Wound-healing assay

LECs were seeded in 6-well plates, grown to confluency, and then scratched across the center using a 200-μL pipette tip. After rinsing with PBS three times, the medium was replenished with FBS-free DMEM to inhibit cell proliferation. Images were taken at the time of wounding (0 h) and at 12 h. Migration was quantified using ImageJ software.

### Cell culture and transfection

Murine bone marrow-derived macrophages (BMDMs) were flushed from the tibia and femurs of 6-week-old WT C57BL/6 mice. The bone marrow cells were flushed out and centrifuged for 5 min at 300×g. After eliminating erythrocytes, the remaining cells were differentiated in Dulbecco's modified Eagle's medium supplemented with 10% fetal bovine serum (Gibco, USA) with 25 ng/mL macrophage colony stimulating factor (M-CSF) for 7 days. The mouse lymph node endothelial cell line (SVEC4-10, LECS) was purchased from the China Center for Type Culture Collection (CCTCC). SVEC4-10 cells were cultured in DMEM supplemented with 10% fetal bovine serum and 1% penicillin/streptomycin. Recombinant CD137L (Cat: HY-P7446) was purchased from MedChemExpress (Shanghai, China).

LECs were transfected with control siRNA, Atg5 and Atg7 siRNA (Ribobio, China) using Lipofectamine iMAX for 24 h. Briefly, cells were seeded into a 24-well plate. The following day the cells were transfected. Lipofectamine and siRNA were separately first mixed with OPTI-MEM at room temperature for 5 min. Subsequently, siRNA was added to Lipofectamine-containing media, incubated for 15 min and then added to cells.

### Tube formation assay

A pre-chilled 48-well cell culture plate was coated with 150 μL of unpolymerized Matrigel (10 mg/mL) and incubated at 37℃ for 1 h. SVEC4-10 cells were harvested and seeded at a density of 10^4^ cells/well. The cells were incubated with or without recombinant CD137L (50 ng/mL) and anti-CD137 antibody (5 μg/well). After approximately 3 hours of incubation at 37℃ in 5 % CO2, cell tube formation was assessed using an inverted fluorescent microscope.

### RNA sequencing and bioinformatics analysis

For RNA-seq, total RNA was extracted from LECs. RNA isolation, library construction, and sequencing were performed on a BGISEQ-2000 (BGI, Shenzhen, China). Fold changes were calculated for all possible comparisons, and a 2-fold cutoff was used to select genes with expression changes. KEGG pathway analysis and GO-P( biological process) was performed using the R package, using significantly differentially expressed genes (p<0.05) as target genes.

### mRFP-GFP-LC3 lentiviral infection

SVEC4-10 cells were infected with lentiviral particles expressing the fusion protein mRFP-GFP-LC3 (Genomeditech, Shanghai, China) for 24 hours. Then, the transfection medium was replaced by culture DMEM supplemented with 10% FBS for 48 h. After treatment, the cells were fixed with 4% formaldehyde. After washing with PBST 3 times, the nuclei were stained with DAPI. The cells were imaged using a confocal fluorescence microscope (Olympus, Japan).

### Statistical analysis

The statistics were first analyzed for normal distribution using the Kolmogorov-Smirnov test. The Continuous variables are showed median (25th-75th percentiles) and categorical variables are showed as a number. Two-tailed unpaired t-tests or chi-squared tests were used for comparisons between two groups, and one-way ANOVA tests were used for three groups. Statistical analysis of data was performed using GraphPad Prism 7. P values < 0.05 were considered statistically significant; *P < 0.05; **P < 0.01; ***P < 0.001, NS = not significant.

## Results

### CD137 expression is increased and correlated with intrarenal lymphangiogenesis and interstitial fibrosis in IgA patients

**Figure 1:**
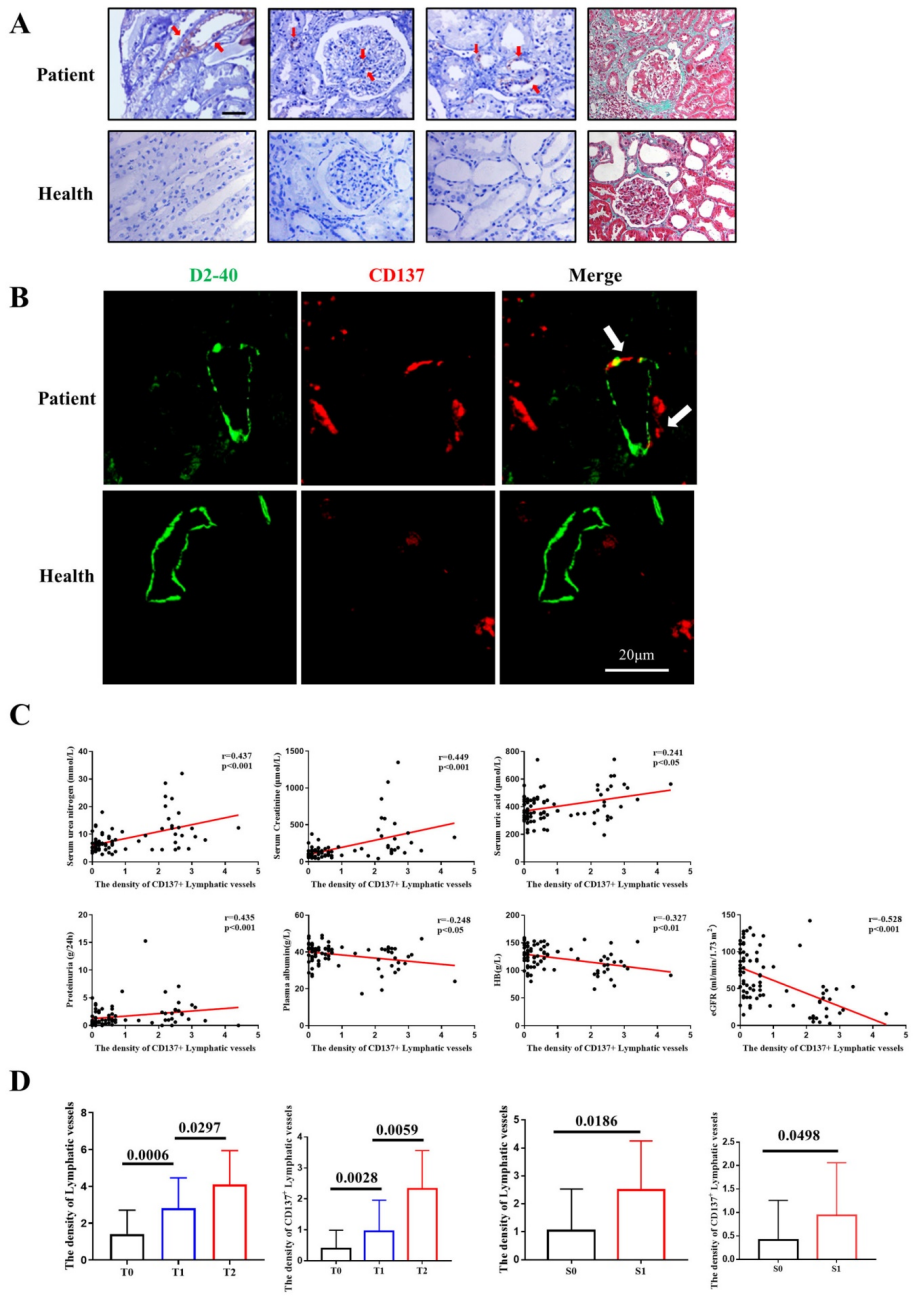
CD137 expression correlated with intrarenal lymphangiogenesis and fibrosis in IgAN. (A) IHC analysis of CD137 expression (40×); Masson's trichome staining showing collagen deposition(40×); (B) Representative images and double-positive analyses for immunofluorescence-labeled D2-40 (green) and CD137 (red) in IgA patients (n=85) and controls (n=5). Scale bar, 20µm. (C) Relationships between CD137^+^ lymphatic vessels in the interstitium and Serum urea nitrogen, Serum Creatinine, Serum uric acid, proteinuria, Plasma albumin, HB and eGFR. HB, hemoglobin; eGFR, estimated glomerular filtration rate; (D) The difference of density of lymphatic vessels and CD137^+^ lymphatic vessels between patients with different T and S grades. T, tubular atrophy/interstitial fibrosis; S, segmental glomerulosclerosis.

CD137 was previously shown to play a key role in T-cell-mediated responses and acts as a costimulatory molecule by binding to its ligand (CD137L). We collected 85 clinical renal biopsy specimens from IgA nephropathy patients in our hospital from January 2020 to April 2020. There were 42 males and 43 females, aged 18 to 65 years. The diagnosis of IgA nephropathy was confirmed by renal pathological examination, and renal biopsy specimens were reassessed blindly by a single pathologist using the Oxford classification. Representative photographs of Masson's trichrome staining are shown in Figure 1A. Immunohistochemistry results showed that CD137 was strikingly expressed in proliferating mesangial cells, injured renal tubular epithelial cells, renal interstitial cells and lymphatic endothelial cells (Figure 1A). Although a previous study reported that inflamed skin contains abundant CD137^+^ lymphatic vessels 27, evidence of CD137^+^ lymphatic endothelial cells during IgA is still absent. We performed immunofluorescence staining using D2-40 as a lymphangiogenesis marker. CD137 expression was costained for D2-40, which was observed as double-positive cells in patients, while healthy individuals showed little or no costaining (Figure 1B).

To test whether the expression of CD137 in lymphatic vessels is related to the clinical parameters of IgA nephropathy patients, we first examined the quantification of colocalization of D2-40/CD137 staining, and the results were semiquantitatively analyzed with ImageJ. We collected clinical data, including age, sex, serum urea nitrogen, serum uric acid, urinary 24 h total protein, HB, serum albumin, serum creatinine, and eGFR, from IgA nephropathy patients. The data analysis shows that the expression level of D2-40 was not correlated with age, sex or serum uric acid but positively correlated with serum creatinine (r=0.438, p<0.001), serum urea nitrogen (r=0.427, p<0.001), and urinary 24 h total protein (r=0.496, p<0.001). The expression levels of D2-40 and CD137 colocalization areas were not correlated with age or sex but were positively correlated with serum creatinine (r=0.499, p<0.001), serum urea nitrogen (r=0.437, p<0.001), serum uric acid (r=0.241, p=0.026), and urinary 24 h total protein (r=0.435, p<0.001). The expression of these indicators was negatively correlated with eGFR (r=-0.473, r=-0.528, p<0.001) (Figure 1C, Table 1).

Moreover, we assessed the relationship between the Oxford classification and the density of CD137-positive lymphatic vessel angiogenesis or total lymphatic vessel angiogenesis. We found that the patients with higher scores for tubular atrophy/interstitial fibrosis showed a higher density of lymphatic vessels. However, the density of CD137-positive lymphatic vessels was better at differentiating the divergence of T1 and T2. In terms of segmental sclerosis, both the density of total lymphatic vessels and CD137-positive lymphatic vessels showed a significant association (Figure 1D).

We further investigated the potential effect of D2-40 and CD137 colocalization area, and ROC curve analysis was used to compare the specificity and sensitivity to predict fibrosis in IgA patients. With the cutoff value of 2.2% in the cohort, patients with a high percentage of colocalization areas exhibited severe tubular interstitial fibrosis (T2, 52.6% versus 5.5%; P<0. 001). There was no difference in mesangial hypercellularity (M1), endocapillary hypercellularity (E1), segmental glomerulosclerosis (S1) or crescents (C2) between the two groups (Table 3). Altogether these results revealed that CD137-positive lymphatic vessels have a close relationship with the prognosis of IgAN.

### Renal CD137^+^ lymphatic vessels are increased in the UUO model

**Figure 2:**
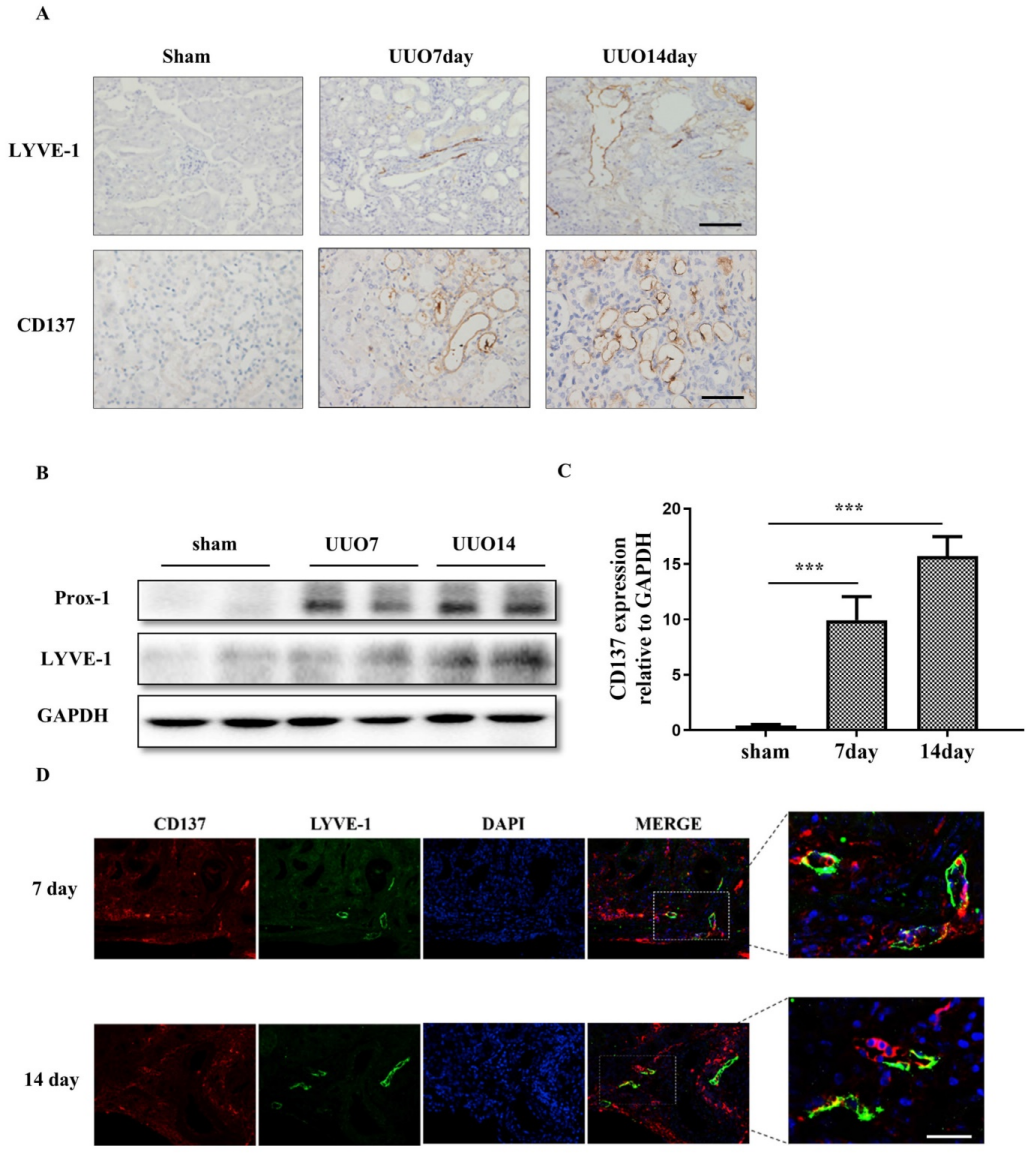
CD137 is increased in the obstructed kidney. (A) IHC staining showing LYVE-1 and CD137 expression in the sham control and UUO mice (400×). The expression levels of Prox-1 and LYVE-1 were detected by Western blot (B), and the expression levels of CD137 were detected by real-time PCR (C). (D) Immunofluorescence staining showing CD137 (red) expression in UUO kidneys in the interstitium on days 7 and 14 and colocalization with LYVE-1 (green) original magnification, ×400. n = 6 per group. The error bars represent the SEM. ***P < 0.001.

To ascertain that damaged kidneys contain abundant CD137^+^ lymphatic vessels, we investigated the expression and location of LYVE-1 and CD137 in a UUO mouse model. Extensive stromal fibrosis was detected by Masson's trichrome staining, and increased CD137 and LYVE-1 expression and infiltrating macrophages were detected by immunohistochemistry (IHC) in mouse kidneys after the operation compared with those of the sham-operated mice; these parameters peaked on day 14 (Figures 2A). Western blotting and real-time PCR results indicated that the expression of lymphangiogenesis markers (Prox-1 and LYVE-1) and CD137 was significantly elevated in the kidneys of UUO mice compared with sham-operated mice, which was consistent with the histopathological results. (Figure 2B, C). Meanwhile, we also observed that some LYVE-1^+^ lymphatic vessels were also positive for CD137 in UUO mice (Figure 2D).

### CD137L induced by macrophages in the Kidney Following Obstructive Injury

**Figure 3:**
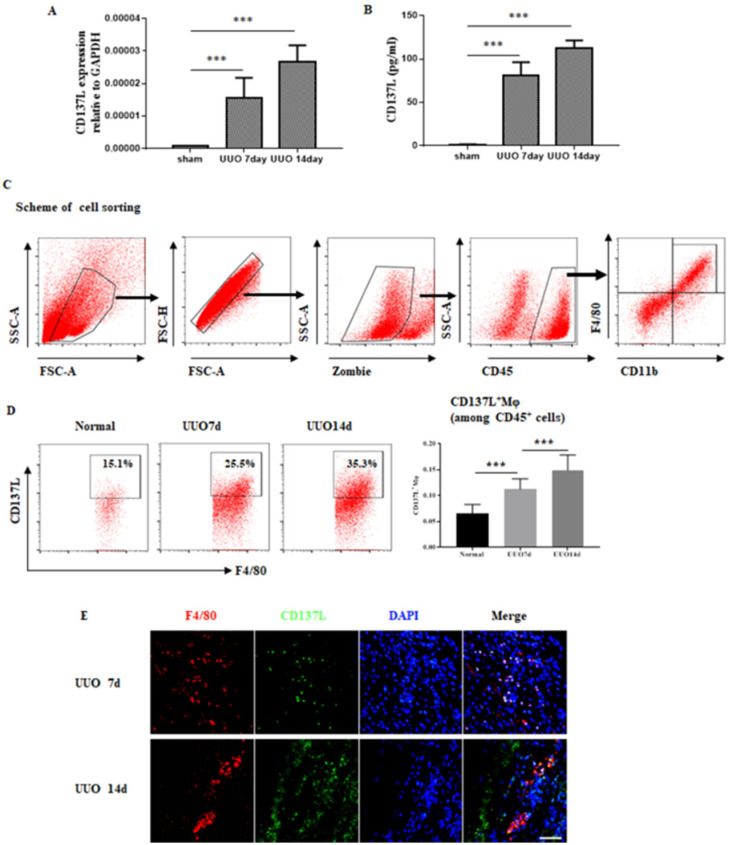
Expression of CD137L on macrophages in UUO mice. Mice were subjected to unilateral ureteral obstruction (UUO) by left ureteral ligation for 7 and 14 days. CD137L mRNA and protein expressions were detected by Real-time PCR (A) and Elisa (B). (C) Scheme of the cell-sorting approach. (D) Flow cytometric analysis and quantification of CD137L^+^ macrophages in sham and UUO mice. kidney cell suspensions from the obstructed kidneys. (E) Immunofluorescence staining showing CD137L (green) expression and colocalization (white arrows) with F4/80 (red) in UUO kidneys. The error bars represent the SEM. ***P < 0.001. Original magnification, Scale bar, 20µm. n= 6 per group. The data were pooled from three independent experiments.

CD137-CD137L interactions have been implicated in acute kidney inflammation, and we proceeded to investigate the role of their interactions during the development of chronic kidney disease. As shown in Figure 3A, renal mRNA levels of CD137L were substantially increased in UUO mice compared with sham control mice. In addition, the serum level of CD137L in UUO mice was significantly increased in the early and late stages compared with that in the serum of sham control mice (Figure 3B). To further define which cells were the major source of CD137L production in UUO kidneys, we performed flow cytometry. The results showed that in the UUO groups, the percentages of CD45^+^CD11b^+^F4/80^+^CD137L^+^macrophages were higher than those of the sham-operated group and increased with the extension of ligation (Figure 3C). Our previous investigation verified that lymphangiogenesis in renal fibrosis arises from macrophages, so we paid more attention to the relationship between macrophages and lymphangiogenesis. Consistent with the FACS data, immunofluorescence staining showed that some macrophages detected in the interstitium colocalized CD137L in UUO mice (Figure 3E).

### VEGF-C stimulates CD137L Expression on macrophages

**Figure 4:**
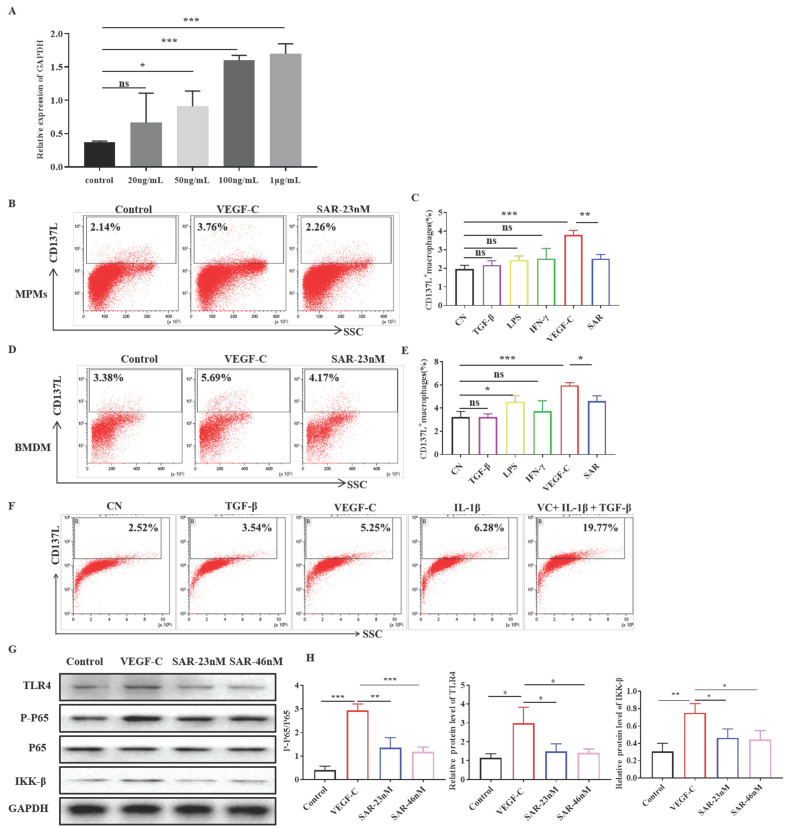
VEGF-C promotes CD137L production in macrophages in vitro. (A)Relative mRNA expression of CD137L was measured in BMDMs treated with VEGF-C (0, 20, 50, 100, 1000 ng/mL) subpopulations by real-time PCR. Flow cytometric analysis and quantification showing the percentages of CD137L^+^ MPMs (B, C) and BMDMs (D, E) with VEGF-C (100 ng/mL) and SAR131675 (23 nM). The data shown were representative FACS profiles. (F) Flow cytometric analysis showing the percentages of CD137L^+^BMDMs with TGF-β(10ng/mL), VEGF-C (100 ng/mL), L-1β (25ng/mL) and mixture of cytokines. The data shown were representative FACS profiles. (G) BMDMs were grown in complete medium and treated with VEGF-C (100 ng/mL) with or without SAR131675 (23 nM, 46 nM) for 48 h. Western blot analysis was performed to measure the expression of TLR4, p-P65, P65, IKK-β and GAPDH. (H) The histograms show the relative intensity for each marker normalized to GAPDH. The error bars represent the SEM. *P< 0.05, **P < 0.01; ***P < 0.001 versus control. The data were pooled from three independent experiments.

We firstly assessed the role of VEGF-C on macrophages by real-time PCR. As shown in Figure 4A, the expression of CD137L was increased in BMDMs treated with VEGF-C (50, 100, 1000 ng/mL). The optimal VEGF-C concentration (100 ng/mL) was used for subsequent experiments. Multiple studies have confirmed that proinflammatory cytokines are abundantly expressed in the fibrosis model. To determine which cytokines induced the expression of CD137L on the surface of macrophages, we used TGF-β, IFN-γ, LPS, and VEGF-C to conduct cell stimulation experiments on peritoneal macrophages (MPMs) and bone marrow-derived macrophages (BMDMs) in vitro (Figure 4B-E). Flow cytometry results showed that only VEGF-C was able to induce the expression of CD137L on both MPMs and BMDMs, and other cytokines had no obvious effect on the expression of MPMs CD137L. Moreover, treatment of macrophages with VEGFR3 inhibitor SAR131675 partially decreased the expression of CD137L induced by VEGF-C. Considering many proinflammatory factors exist simultaneously in vivo, we used a mixture of proinflammatory cytokines to stimulate macrophages. The results showed that the expression of CD137L on macrophages increased sharply induced by VEGF-C, TGF-β and IL-1β (Figure 4F). Our data indicated that VEGF has synergism with other inflammatory factors on macrophages during inflammatory conditions in vivo. And we also confirmed the increased renal VEGV-C expression in UUO mice Supplement Figures 2.

To investigate the mechanism of CD137 expression, Western blotting was utilized to detect the TLR4 pathway. As shown in Figure 4G, treatment with VEGF-C increased the levels of TLR4, phosphorylated P65 and IKK-β in macrophages. In addition, the different concentrations of SAR131675 inhibited TLR4 activation and decreased the expression of CD137L induced by VEGF-C. Finally, we demonstrated that the increases observed in CD137 expression in mouse macrophages were dependent on VEGF-C/VEGFR-3 signaling pathway.

### The biological effects of CD137L on LECs in vitro

**Figure 5:**
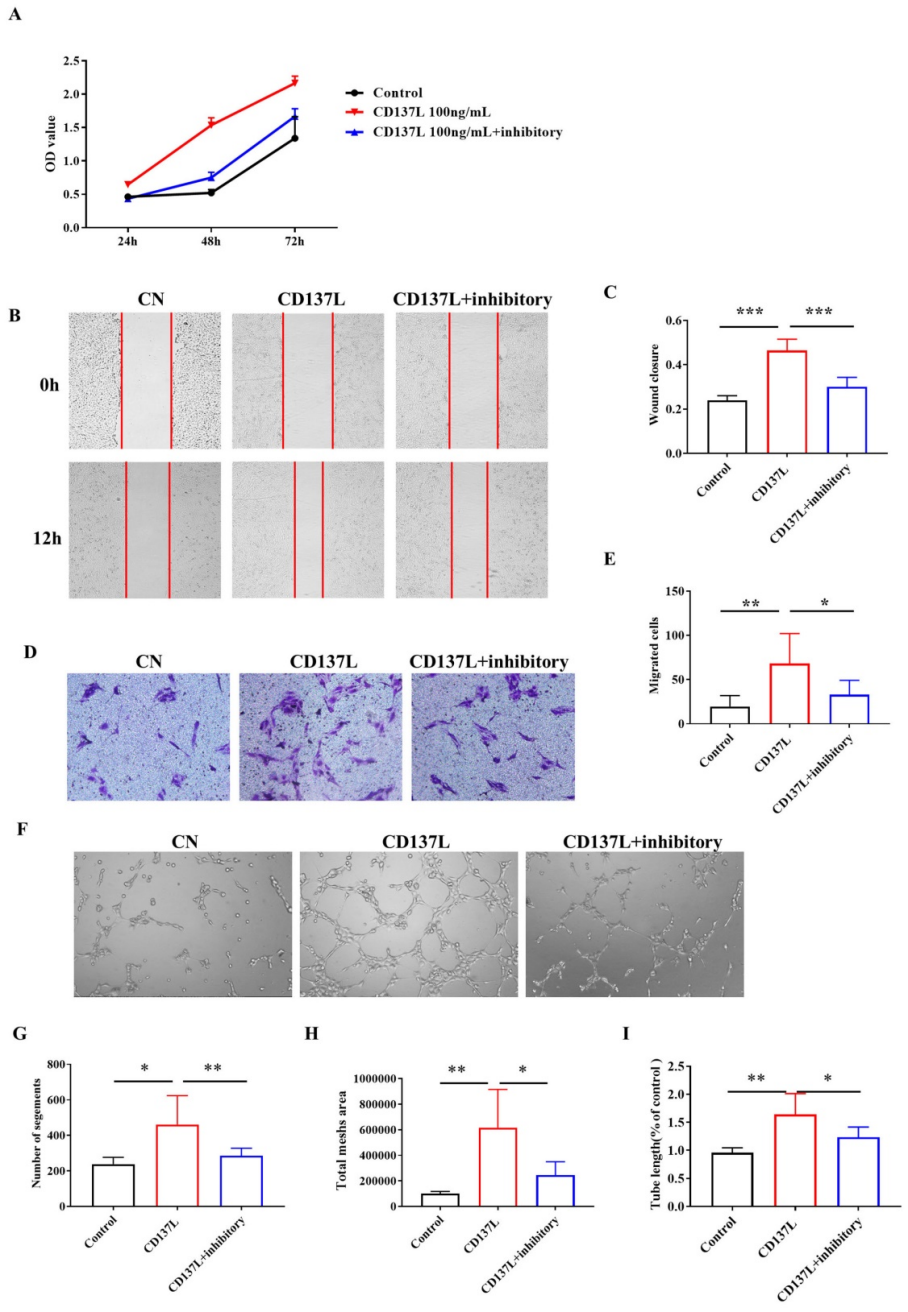
CD137L promotes the proliferation, migration and tube formation of lymphatic endothelial cells. (A) The proliferation of LECs treated with CD137L or CD137L and inhibitory for 24 h, 48 h, and 72 h was examined by CCK-8 assay. The migration of LECs was measured with the cell scratch test (B) and Transwell migration assay (D) after CD137L or CD137L and inhibitory treatment for 12 h. The histograms represent migrated (C, E) cells per field. (F) A tube formation assay was used to test the lymphangiogenesis capacity of LECs after CD137L or CD137L and inhibitory treatment for 3 h (image magnification, 100×). The histograms represent the branch number per field. (G, H, I). The error bars represent the SEM. *P< 0.05, **P < 0.01; ***P < 0.001. The data were pooled from three independent experiments.

Given that both CD137 on LECs and CD137L expression are elevated in the kidneys of UUO mice, we then proceeded to investigate the biological effects of CD137L on LECs in vitro. As shown in Figure 5A, increased cell growth was observed in SVEC4-10 cells after 48 h of culture in CD137L-containing media. The population-doubling time of SVEC4-10 cells with CD137L was significantly shorter than that of the control cell lines. The scratch test showed that CD137L on LECs for 12 h significantly increased the migration distance of LECs compared with that of the control group (Figure 5B). The Transwell chamber results showed that the number of migrating LECs upon culturing for 24 h with the addition of CD137L significantly increased 2- to 3-fold (Figure 5D, P < 0.001). Subsequently, the tube formation assay of LECs showed that CD137L also increased tube formation (Figure 5E). Moreover, treatment of LECs with CD137 inhibitory antibody partially inhibited the proliferation and decreased the migration distance and the number of migrating cells to the lower chamber and tube formation induced by CD137L. These findings indicate that the biological effects of CD137L on LECs are largely dependent on the expression of its receptor, CD137.

### Transcriptome analysis of LECs after CD137L treatment

**Figure 6:**
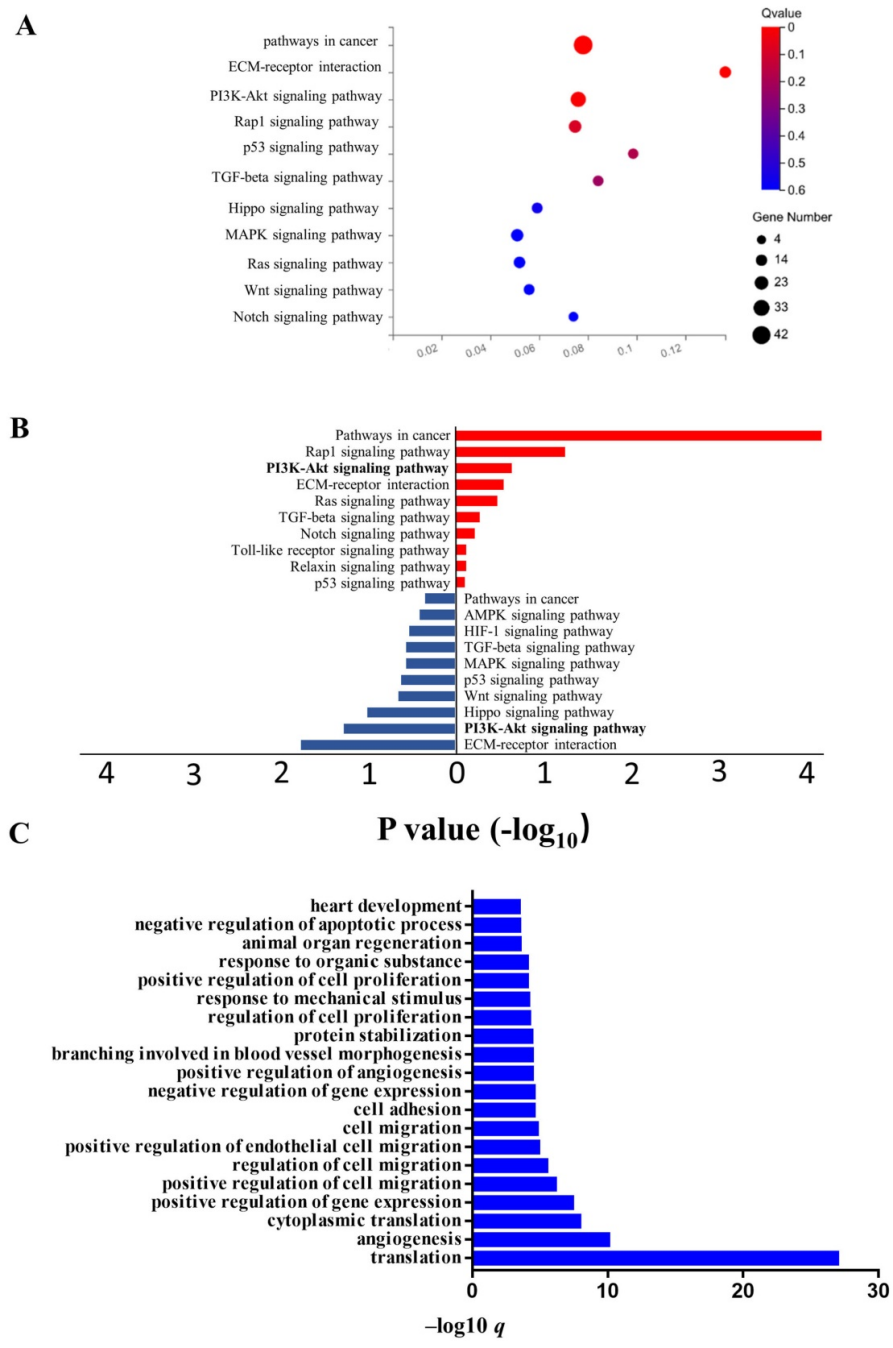
Transcriptome analysis of LECs after CD137L treatment (A) Eleven significantly activated KEGG pathway in SVEC4-10 cell line after treatment with CD137L. (B) Enriched KEGG pathway for differentially expressed genes in SVEC4-10 cells with or without CD137L treatment. The terms enriched from upregulated genes in CD137L treated SVEC4-10 cells are marked by red, and terms enriched from down regulated genes are marked by blue. (FDR≤0.01) (C) Top20 significantly activated biological processes in SVEC4-10 cell line after treatment with CD137L, as indicated in GO analysis (FDR≤0.01). GO, gene ontology; DEGs, differentially expressed genes; FDR, false discovery rate; KEGG, Kyoto Encyclopedia of Genes and Genomes.

It is clear from the above results that the CD137/CD137L network promotes LECs to exert its multiple functions. To further characterize the mechanism of LECs after CD137L treatment, we performed RNA sequencing (RNA-seq) on SVEC4-10 cells to analyze their whole transcriptomic profiles. Kyoto Encyclopedia of Genes (Figure 6A) and Genomes (KEGG) analysis (Figure 6A) showed that one of the most prominent pathways in LECs upon CD137L treatment was the PI3K-Akt signaling pathway. Our results showed that CD137L remarkably decreased the phosphorylation levels of PI3K, Akt, and mTOR. GO enrichment analysis indicated that cell adhesion progress, cell migration progress and angiogenesis progress were activated in LECs after CD137L treatment (Figure 6C). Our data showed that CD137L treatment accelerated LEC migration and tube formation. However, the capacity of recombinant CD137L treatment was obviously suppressed by CD137 inhibitory antibody and autophagy inhibitor 3-MA.

### CD137L induced the autophagy pathway via inactivation of the PI3K/AKT/mTOR signaling pathway

**Figure 7:**
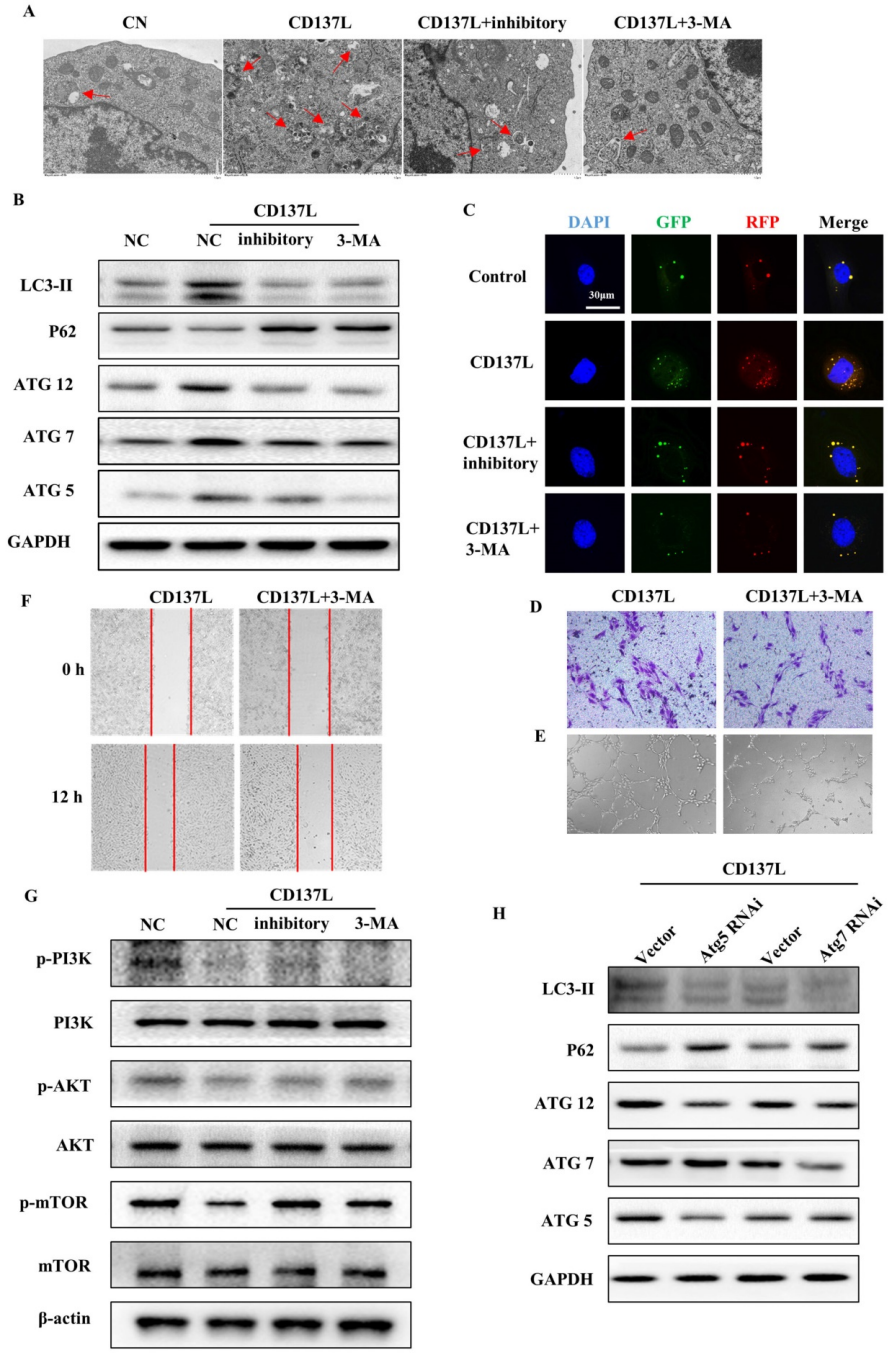
CD137L induced autophagy of lymphatic endothelial cells via mTOR pathway. (A) Representative electron micrographs of CD137L-induced autophagosome in SVEC4-10 cells that were either stimulated with inhibitory, or with 3-MA for 24 h. (B) The protein expression levels of autophagy-associated proteins (Atg5, Atg7 and Atg12) and autophagic substrates (LC3 and p62) were analyzed in SVEC4-10 cells after CD137L, inhibitory or 3-MA treatment for 24 h by Western blot. (C) SVEC4-10 cells were infected with RFP-GFP-LC3-expressing lentivirus. Cells were untreated or treated with CD137L, inhibitory or 3-MA for 24 h. Fluorescence was examined by confocal microscopy. Transwell migration assay (D), scratch test (E) and tube formation assay (F) stimulated with CD137L for 12 h, and with or without 3-MA treatment. (G) The protein expression levels of phosphorylated PI3K, Akt and mTOR were analyzed in SVEC4-10 cells after CD137L or inhibitory or 3-MA treatment for 24 h by Western blot. (H) SVEC4-10 cells were knocked down Atg5 and Atg7 by using siRNAs, and stimulated with CD137L for 24h, the protein expression levels of autophagy-associated proteins (Atg5, Atg7 and Atg12) and autophagic substrates (LC3 and p62) were detected by Western blotting.

Autophagy is initiated as a protective response to environmental stressors, and the excessive activation of autophagy can lead to the pathogenesis of cancer, cardiovascular and kidney disease. To demonstrate whether autophagy is involved in renal fibrosis, SVEC4-10 cells were treated with CD137L or CD137L plus inhibition, and the ultrastructure of the cells was analyzed by electron microscopy. The number of autophagic vesicles was also significantly increased in SVEC4-10 cells treated with CD137L for 24 h, and the number of autophagic vesicles was reduced in cells treated with CD137L and inhibited for 24 h (Figure 7A). Moreover, we measured the expression of the autophagy-related proteins LC3II, P62, Atg5, Atg7 and Atg12 in LECs treated with CD137L. The expression levels of LC3II, Atg5, Atg7, and Atg12 were increased, and P62 expression was decreased. Cotreatment of LECs with inhibitory or 3-MA inhibited CD137L-induced autophagy (Figure 7B). Autophagy is a dynamic process and we try to clarify the effects CD137L exerts on LECs. We transfected SVEC4-10 cells using GFP-RFP-LC3 lentiviral particles and observed under confocal microscopy view. The autolysosomes were labeled with red dots, and the autophagosomes were both labeled with red dots and green dots. After incubating with recombinant CD137L protein for 24h, the number of autophagosomes (yellow dots, a merge of red dots and green dots) and autolysosomes (green dots) increased remarkably, while the number of both autophagosomes and autolysosomes did not increase significantly after pretreated with CD137 inhibitory. The process of autophagy was inhibited after treating with CD137L and 3-MA (10 mM) (Figure 7C). Moreover, the inhibition of CD137L-induced autophagy of LECs with 3-MA led to inhibited proliferation and decreased migration distance and the number of migrating cells to the lower chamber and tube formation induced by CD137L (Figure 7D, E and F).

To investigate the mechanism by which CD137L induced autophagy, Western blotting was utilized to detect the PI3K/AKT/mTOR pathway. As shown in Figure 7G, treatment with CD137L decreased the levels of phosphorylated PI3K, Akt and mTOR in LECs. Cotreatment of LECs with inhibitory or 3-MA attenuated CD137L-induced signaling pathway inactivation. We next inhibited autophagy by Atg5 siRNA or Atg7 siRNA. The results showed that LECs transfected with siRNA specifically targeting Atg5 or Atg7 suppressed CD137L-induced autophagy, suggesting the requirement of these genes in CD137L-induced autophagy (Figure 7H).

## Discussion

Our group previously reported that renal macrophage infiltration and lymphangiogenesis were strongly associated with the progression of fibrosis 7. In this study, we further analyzed the correlation between CD137^+^ lymphatic vessels and clinical parameters of IgA nephropathy patients and explored the influence of CD137^+^ lymphangiogenesis on the progression of IgA nephropathy to renal fibrosis. Moreover, compared with lymphatic vessel densities, CD137^+^ lymphatic vessel densities were more associated with tubular atrophy/interstitial fibrosis. Then, we observed that CD137^+^ lymphatic vessels were involved in the development of IgA nephropathy and positively correlated with the severity of the disease. CD137 is mainly expressed in T lymphocytes and natural killer (NK) cells and in noninflammatory cells such as endothelial cells 24-26. Teijeira and colleagues have provided compelling evidence for inflammation-dependent expression of CD137 on the surface of LECs in vitro. CD137^+^ lymphatic vessels were found in the dermis of patients diagnosed with inflamed or infected skin ulcers, while they were lacking in the lymphatic vessels of noninflamed tissues 27. Our research also confirmed that CD137 was strikingly expressed in lymphatic endothelial cells in IgA patients. Similar results were confirmed in the UUO mice model.

During renal fibrosis, macrophages are actively involved in promoting lymphangiogenesis in two ways. One is to transdifferentiate into lymphatic vessels in human kidney transplants 28, 29, in a UUO mouse model 30, and in murine tumor models 31. Another way is to secrete prolymphangiogenic growth factors such as VEGF-C, VEGF-D, and VEGF-A in response to inflammatory mediators 32-35. In op/op mice, lymphatic vessel development is delayed due to the lack of macrophages, which demonstrates that macrophages seem to be important sources of lymphangiogenic factors 36. In this study, we found that only VEGF-C, but not inflammatory cytokines (IFN-γ and LPS), could stimulate CD137L expression on both peritoneal macrophages (MPMs) and bone marrow-derived macrophages (BMDMs). This reason can be explained by the heterogeneity of macrophages. BMDMs, as circulating macrophages, are ex vivo differentiated from stem cells and are more responsive to environments. MPMs, as resident macrophages, are minimally replaced by blood monocytes under homeostatic conditions 37,38. BMDM responses are a function of intrinsic differences, while MPM responses reflect their differentiation in the context of the whole animal. In previous studies, we also noticed a difference between resident and circulating macrophages. We found that the level of local C3 mostly secreted by resident macrophages was highly correlated with RF, while glomerular C3 deposition secreted by infiltrating macrophages was also associated with the severity of renal lesions 39. These results suggest that resident macrophages not only phagocytose the environment but also respond rapidly to the environment and release cytokines.

VEGF-C is a classic lymphatic growth factor produced by circulating macrophages, podocytes and tubule epithelial cells 40-42. It promotes expansion and reconstruction of the lymphatic capillary net through activation of VEGFR-3 on LECs, leading to lymphangiogenesis in chronic kidney diseases. In acute kidney injury, pathogen-associated molecular patterns (PAMPs) and damage-associated molecular patterns (DAMPs) activate resident macrophages and kidney parenchymal cells to secrete inflammatory cytokines and chemokines 43,44. Our research found that at the beginning of injury, damaged parenchymal cells released VEGF-C and directly activated resident macrophages through TLR4, which mediated rapid activation of NF-κB and thereby stimulated the expression of CD137L. We also confirmed that VEGF-C could activate circulating macrophages to stimulate the expression of CD137L. Notably, resident and infiltrating macrophages may indirectly and directly participate in different phases of kidney disease by VEGF-C.

CD137-CD137L signaling participated in multiple stages of inflammation. CD137 is expressed on activated T cells, B cells, monocytes and NK cells. Recently, CD137 expression was also confirmed on the dermal lymphatic endothelial cells and blood endothelial cells of inflamed tissue samples, in which cross-linking with CD137L resulted in NF-κB nuclear translocation, followed by increased production of VCAM and CCL21^27^, ^45^-^47^. However, there are no reports regarding the expression and functionality of CD137 in renal lymphatic endothelial cells (LECs). In ischemic acute kidney injury, increased CD137 on infiltrating NK cells induces recruitment of neutrophils by stimulating the CD137L signaling pathway in TECs, which leads to their production of CXCL1 and CXCL2 through increased phosphorylation of p38 and JNK kinases and affects the degradation rate of IκBα 21. Similar to IRI, we found that CD137 was upregulated on lymphatic endothelial cells in IgA nephropathy patients, and the density of CD137^+^ lymphangiogenesis was positively correlated with renal interstitial fibrosis.

Autophagy dysfunction is involved in the pathogenesis of various renal diseases 48-51. In patients with CKD, autophagy activities may increase to remove uremic toxin-induced injured components, which may have a protective role against CKD progression 52. However, in a mouse UUO model and TGF-β1-treated PTECs, the effects of autophagy in renal interstitial fibrosis were controversial. Under this condition, protein kinase C (PKC)-α, by stimulating autophagic flux, promoted autophagy and apoptosis in renal tubules and drove renal interstitial fibrosis 53. In contrast, other studies show that autophagy deficiency in proximal tubules leads to G2/M cell cycle arrest and increased production of collagen I, which accelerates renal interstitial fibrosis 54. Inhibition of autophagy by 3-methyladenine promoted fibrosis by inducing tubular cell apoptosis in a rat model of UUO 55, 56. The inconsistency of these results may be related to many types of renal resident cells, including podocytes, mesangial cells, glomerular endothelial cells, renal tubular epithelial cells and lymphatic endothelial cells. The role of autophagy in lymphatic endothelial cells is poorly understood. In this study, we found that the induction of autophagy by CD137L in lymphatic endothelial cells was mediated by the PI3K/AKT/mTOR signaling pathway. Enhanced autophagy led to increased proliferation, migration and tube formation of lymphatic endothelial cells, which ultimately formed lymphangiogenesis and promoted renal interstitial fibrosis.

Combined with the data obtained from patient samples, relevant animal experiments and cell models, our study defines a novel mechanism by which CD137^+^ lymphatic vessels participate in renal fibrosis. In response to injury, CD137L secretion by macrophages leads to LEC autophagy, which further drives lymphangiogenesis responses. Our present results add to the growing body of evidence supporting lymphangiogenesis as a potential therapeutic approach for obstructive nephropathy.

## Supplementary Material

Supplementary figures and tables.

## Figures and Tables

**Figure 8:**
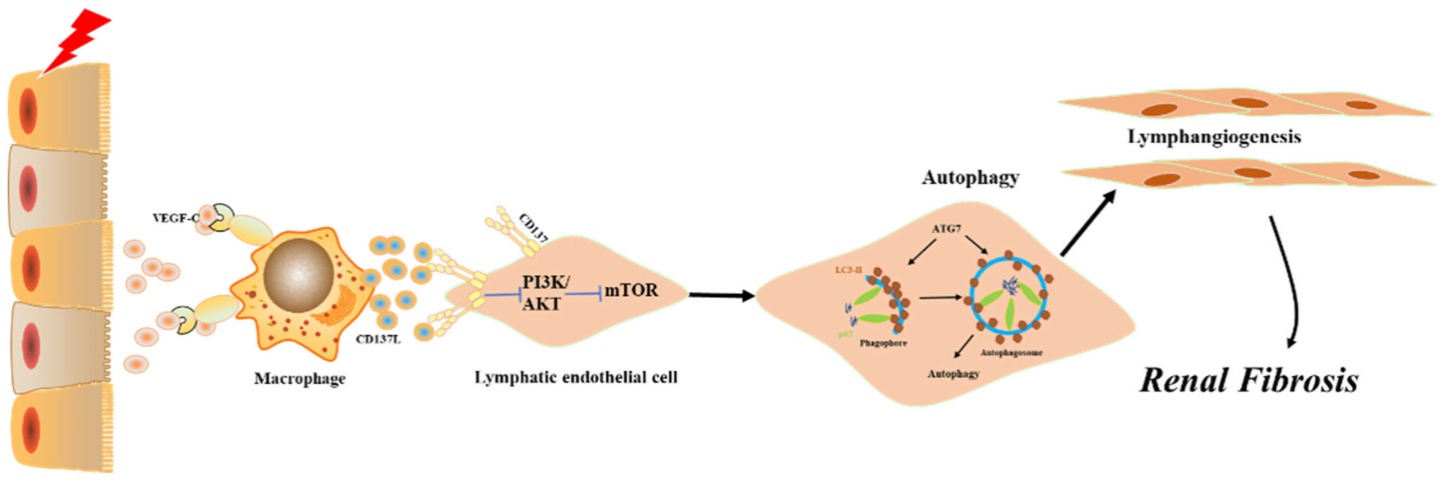
Model summarizing the role of CD137^+^ lymphatic vessels participate in renal fibrosis. CD137L secretion by macrophages leads to LEC autophagy, involving the inactivation of mTOR and PI3K signaling pathway.

**Table 1:** Demographic and clinical parameters between patients with D2-40 and CD137^+^D2-40 expression.

Variables	ALL(n=85)	D2-40	CD137^+^D2-40
Male gender (%)	42.0(49.4%)	-0.151	-0.092
Age (years)	34.0(28.0-44.0)	-0.053	-0.075
Serum Creatinine (μmol/L)	119.0(83.0-189.0)	0.438^c^	0.499^c^
Serum urea nitrogen (mmol/L)	6.64(4.65-9.95)	0.427^c^	0.437^c^
Serum uric acid (μmol/L)	375.0(325.0-454.0)	0.183	0.241^a^
eGFR (ml/min/1.73 m2)	54.2(36.15-90.25)	-0.473^c^	-0.528^c^
Proteinuria (g/24h)	1.0(0.59-2.49)	0.496^c^	0.435^c^
Plasma albumin	40.1(35.8-41.95)	-0.284^b^	-0.248^a^
HB (g/dL)	123.5(106.25-142.5)	-0.352^b^	-0.327^b^

Data are presented as a percentage or median (25^th^--75th percentiles). Values of the lymphatic vessels are presented as the number of cells per millimeter squared. ^a^P<0.05; ^b^P<0.01; ^c^P<0.001. eGFR: estimated glomerular filtration rate. HB: hemoglobin.

**Table 2:** Demographic and clinical parameters between patients with low or high levels of CD137^+^D2-40 expression.

Variables	ALL(n=85)	CD137^+^D2-40^low^ (n=61)	CD137^+^D2-40^high^ (n=24)	*p*
Male gender (%)	42.0(49.4%)	31(50.8%)	11(45.8%)	ns
Age (years)	34.0(28.0-44.0)	34.0 (28.5-45.0)	33.5 (27.0-37.5)	ns
Serum Creatinine (μmol/L)	119.0(83.0-189.0)	98.0 (76.5-148.0)	237.5 (139.8-496.3)	<0.001
Serum urea nitrogen (mmol/L)	6.64(4.65-9.95)	6.1 (4.4-7.4)	12.05 (5.52-17.18)	<0.001
Serum uric acid (μmol/L)	375.0(325.0-454.0)	359.0 (318.9-437.5)	449.0 (361.9-555.0)	0.0022
eGFR (ml/min/1.73 m2)	54.2(36.15-90.25)	72.1 (46.7-101.4)	27.2 (10.68-48.98)	<0.001
Proteinuria (g/24h)	1.0(0.59-2.49)	0.51 (0.29-1.06)	1.0 (0.74-2.29)	0.0006
Plasma albumin (g/L)	40.1(35.8-41.95)	40.8(36.83-42.35)	38.0(31.3-41.75)	0.0294
HB (g/dL)	123.5(106.25-142.5)	129.0(116.25-144.00)	108.5(93.5-124.0)	0.0001

**Table 3:** Histopathologic features from renal biopsies between patients with low or high levels of CD137^+^D2-40 expression.

Variables	ALL(n=74)	CD137^+^D2-40^low^ (n=55)	CD137^+^D2-40^high^ (n=19)	*p*
Mesangial hypercellularity				
M0	60(81.1%)	43(78.2%)	17(89.5%)	ns
M1	14(18.9)	12(21.8%)	2(10.5%)	
Segmental glomerulosclerosis				
S0	7(9.5%)	6(10.9%)	1(5.3%)	ns
S1	67(90.5%)	49(89.1%)	18(94.7%)	
Endocapillary hypercellularity				
E0	6(8.1%)	4(7.3%)	2(10.5%)	ns
E1	68(91.9%)	51(92.7%)	17(89.5%)	
Tubular intersititial fibrosis				
T0	40(54.1%)	37(67.3%)	3(15.8%)	<0.001
T1	21(28.3%)	15(27.3%)	6(31.6%)	
T2	13(17.6%)	3(5.5.%)	10(52.6%)	
Crescents				
C0	42(56.8%)	32(58.2%)	10(52.6%)	ns
C1	28(37.8%)	20(36.4%)	8(42.1%)	
C2	4(5.4%)	3(5.5%)	1(5.3%)	

Data are presented as percentages. Pathologic features were scored using the Oxford classification.
