# Supplementary material for: CD137L-macrophage induce lymphatic endothelial cells autophagy to promote lymphangiogenesis in renal fibrosis
Source: Int J Biol Sci. 2022 Jan 1;18(3):1171–87. doi: 10.7150/ijbs.66781 (PMC8771854; doi:10.7150/ijbs.66781)
Supplement: Supplementary file 1 — Supplementary figures and tables. [file ijbsv18p1171s1.pdf]

Supplement Figure 1. LYVE-1 antibody inhibits lymphantiogesis and renal fibrosis in UUO. Mice were subjected to unilateral ureteral obstruction (UUO) by left ureteral ligation for 7 and 14 days. UUO mice were subcutaneously injected with antibody and control IgG. On days 7 and 14, mice were sacrificed, and the left kidneys were collected.

The expression levels of lymphangiogenesis markers (LYVE-1 and Prox-1) and fibrotic markers (α-SMA, Collagen I, and PDGFR-β) in Sham and UUO mouse kidneys were detected by Western blot. n = 6 per group.The error bars represent the SEM. **$P < 0.01$ vs. sham operation. n= 6 per group. The data were pooled from three independent experiments.

Supplement Figure 2. The expression of VEGF-C is increased in UUO mice. (A) IHC staining showing VEGF-C expression in the sham control and UUO mice. The expression level of VEGF-C was detected by Western blot (B). (C) Immunofluorescence staining showing VEGF-C (red) expression in UUO kidneys; LTL (green) identified proximal tubules; DBA (green) identified distal convoluted tubules, original magnification, ×400. n = 6 per group. The error bars represent the SEM.
